# Anti-Mucin1 Aptamer-Conjugated Chitosan Nanoparticles for Targeted Co-Delivery of Docetaxel and IGF-1R siRNA to SKBR3 Metastatic Breast Cancer Cells

**DOI:** 10.29252/.23.1.21

**Published:** 2019-01

**Authors:** Reza Jafari, Naime Majidi Zolbanin, Jafar Majidi, Fatemeh Atyabi, Mehdi Yousefi, Farhad Jadidi-Niaragh, Leili Aghebati-Maleki, Dariush Shanehbandi, Mohammad-Sadegh Soltani Zangbar, Houshang Rafatpanah

**Affiliations:** 1 Department of Immunology, Faculty of Medicine, Mashhad University of Medical Sciences, Mashhad, Iran; 2 Immunology Research Center, Division of Inflammation and Inflammatory Diseases, Mashhad University of Medical Sciences, Mashhad, Iran; 3 Department of Pharmacology and Toxicology, School of Pharmacy, Tabriz University of Medical Sciences, Tabriz, Iran; 4 Drug Applied Research Center, Tabriz University of Medical Sciences, Tabriz, Iran; 5 Immunology Research Center, Tabriz University of Medical Sciences, Tabriz, Iran; 6 Department of Immunology, School of Medicine, Tabriz University of Medical Sciences, Tabriz, Iran; 7 Department of Pharmaceutics, Faculty of Pharmacy, Tehran University of Medical Sciences, Tehran, Iran; 8 Nanotechnology Research Center, Faculty of Pharmacy, Tehran University of Medical Sciences, Tehran, Iran

**Keywords:** Breast cancer therapy, Chitosan nanoparticles, Docetaxel, IGF-1R siRNA, MUC1 aptamer

## Abstract

**Background:**

Targeted co-delivery of siRNA and a chemotherapeutic drug is an attractive approach to cancer drug design and treatment. This study was carried out to design an anti-Mucin1 aptamer (Apt)-conjugated chitosan nanoparticle (NP) for targeted co-delivery of insulin-like growth factor receptor 1 (IGF-1R) Silencer siRNA and docetaxel (DTX) to SKBR3 cells.

**Methods:**

Characterization of nano-drugs, cellular uptake of NPs, cell viability, and gene expression studies were evaluated based on metastatic breast cancer cells.

**Results:**

The results of this study showed that NPs had spherical and smooth morphology with 110-118 nm in size and had positive zeta potential (12-14 mV). siRNA and DTX were considerably loaded into NPs. The appropriate conjugation of the Apt to the NPs was affirmed by gel electrophoresis. The Apt-conjugated NPs were observed to enhance the cellular uptake of NPs into the SKBR3 cells. Although the combination treatment significantly decreased the cell viability of SKBR3 cells, the augmentative effect was observed when Apt was conjugated to NPs. Furthermore, Apt-conjugated NPs dramatically reduced the genetic expression of IGF-1R, signal transducers and activators of transcription 3 (STAT3), matrix metalloproteinases (MMP9), and vascular growth factor (VEGF).

**Conclusion:**

The targeted NPs may augment the targeting of pathways involved in tumorigenesis and metastasis of breast cancer. Therefore, more animal model experiments are needed to further clarify the efficacy and safety of this functionalized nanodrug.

## INTRODUCTION

One of the therapeutic strategies for the treatment of metastatic breast cancer is using the combination of two or more anti-cancer agents. For synergistic therapeutic effect, co-delivery of drug and gene using nanocarriers has been extensively investigated[1].

Gene silencing by small interfering RNA (siRNA) is a progressive therapeutic approach for treating metastatic breast cancer. However, the low cellular uptake and short extracellular half-life of siRNA are the biggest challenges in this medicinal application[2]. To overcome this challenge, several nanocarriers have been developed in recent years. Chitosan (CH)-based nanoparticles (NPs) are derived from natural chitin and are suitable carriers for siRNA and drug delivery due to their biocompatibility, biodegradability, low-toxicity, and negligible immunogenicity. Also, due to the positive charge of CH, negatively charged siRNA is efficiently loaded into NPs, and subsequent cellular uptake of nanodrug is augmented[3,4].

The insulin-like growth factor receptor 1 (IGF-1R) is a tyrosine kinase receptor that is essential for normal cell growth and survival. It has been demonstrated that IGF-1R signaling is important in tumor growth, development, and its metastasis. The IGF1-R has vast cross-talk with other signaling pathways, such as the epidermal growth factor receptor and human epidermal growth factor receptor 2[5]. These receptors are up-regulated in metastatic breast cancer cells and can activate important transcriptional factors known as signal transducers and activators of transcription 3 (STAT3). Activated STAT3 up-regulates gene expression, such as matrix metalloproteinases (MMPs) and vascular endothelial growth factor (VEGF), which are critical for invasion and angiogenesis, respectively[6]. Accordingly, studies on the inhibition of IGF-1R expression by siRNA in various cancer cells have been indicated that IGF1-R silencing significantly decreases tumorigenicity and metastasis[7,8].

Docetaxel (Taxotere^®^, DTX) is a cytotoxic anti-cancer drug approved for the treatment of metastatic breast cancer. DTX disrupts the formation of mitotic microtubules and also triggers apoptosis in breast cancer[9]. Similar to other chemotherapeutic drugs, DTX has many adverse reactions, such as allergic reactions, immunosuppression, and hematologic toxicities[10]. For reducing toxicity and enhancing the delivery of DTX to cancer cells, CH-based NPs have been widely studied[11].

Aptamer (Apt) is a synthetic oligonucleotide or peptide molecule that specifically binds to various biomolecular ligands with high affinity and is analogous to monoclonal antibody[12]. These smart ligands may be preferred to monoclonal antibodies because of their affordable production cost, negligible immunogenicity, and small size[13]. Mucin1 (MUC1) is a glycoprotein that is regularly expressed in normal breast epithelial cells. It is aberrantly overexpressed in breast cancer cells and therefore is known as a tumor-associated antigen. Thus, MUC1 Apt can be used for targeted drug delivery to breast cancer cells[14]. In the present study, we designed the CH-based nanocarrier for co-delivery of DTX and IGF-1R siRNA, and subsequently, MUC1 Apt was conjugated to the NPs for evaluating cell viability and genes expression in metastatic breast cancer cells.

## MATERIALS AND METHODS

### Reagents and cell lines

The SKBR3 and CHO cell lines were obtained from the National Cell Bank of Iran (Pasteur Institute of Iran, Tehran, Iran), DTX from AQVida^®^ (Hamburg, Germany), and human IGF-1Rα/β siRNA (sc-29358) and scrambled siRNA (sc-37007) were obtained from Santa Cruz Biotechnology^®^ (Santa Cruz, CA, USA). The MUC1-specific DNA Apt (5’-Amino-C6-GGG AGA CAA GAA TAA ACG CTC AAG AAG TGA AAA TGA CAG AAC ACA ACA TTC GAC AGG AGG CTC ACA ACA GGC-3’) was purchased from TAG A/S^®^ (Copenhagen, Denmark). 1-Ethyl-3-(3-dimethylaminopropyl) carbodiimide (EDC), N-hydroxysuccinimide (NHS), membrane dialysis bag (12 kDa cut-off), and 3-(4,5-Dimethyl-2-thiazolyl)-2,5-diphenyl-2H-tetrazolium bromide (MTT) were procured from Merck^®^ (Hohenbrunn, Germany). RPMI-1640, Trypsin-EDTA (0.25%), and fetal bovine serum (FBS) were obtained from Gibco^®^ (Gibco, Canada), green fluorescence protein (GFP)-containing plasmid (pEGFP-N1 vector) from Clontech Laboratories^®^ (CA, USA), and RNX-Plus from Sinaclon (Tehran, Iran). All other reagents were of analytical grade.

### Preparation of nanodrugs

The preparation of nanodrugs was done using the previously described method with some modifications[15]. The yellowish lyophilized depolymerized CH with a molecular weight of 50 kDa (0.1% w/v) was dissolved in diethyl pyrocarbonate-treated water under magnetic stirring for two hours. Negatively charged carboxymethyl dextran (CMD) was used to form an electrostatic interaction with positively charged CH. Therefore, CMD solution (0.1% w/v) was prepared by dissolving CMD in diethyl pyrocarbonate -treated water (pH 7). Subsequently, 3 µl of IGF-1Rα/β siRNA (19 µg/µl) and 5 µl of DTX (50 µg/ml) were added to 1.2 ml of CMD. Eventually, the obtained aqueous solution was added dropwise to 1 ml of CH solution under gentle magnetic stirring. The nanodrugs were then incubated in the dark at room temperature for 30 min for further analysis.

### *In vitro* characterization of NPs

#### Particle size, polydispersity index (PDI), and zeta potential

The particle size, PDI, and zeta potential of freshly prepared NPs were determined by the Photon Correlation Spectroscopy using Zetasizer Nano ZS (Malvern Instruments Ltd., Malvern, Worcestershire, UK) at a wavelength of 633 nm with an angle detection of 90°. Each sample was measured in triplicate at 25 °C.

#### Morphological analysis

The surface morphology of the freshly prepared NPs was investigated using a transmission electron microscope (TEM, LE-O906 Zeiss^®^, Oberkochen, Germany). A drop of NPs was immobilized on the copper micro-grid and was stained with 3% w/v phosphor tungstic acid. After the evaporation of the sample at room temperature, NPs were examined under the TEM.

### Confirmation of siRNA entrapment into NPs

The confirmation of the siRNA-loaded NPs was assessed by electrophoresis on a 2% agarose gel. Naked siRNA and unloaded CH NPs were used as positive and negative controls, respectively.

### Measurement of drug and siRNA loading efficiency

The loading efficiency of IGF-1R siRNA and DTX was measured using a UV-Vis spectrophotometer (Nanodrop ® 2000, Thermo Scientific, Worcester, MA, USA) at 260 and 230 nm, respectively. The optical density (OD) of the free siRNA was obtained after the centrifugation of CH + CMD + siRNA at 13600 ×g for 20 min. Furthermore, the OD of the free DTX was obtained after the centrifugation of CH + CMD + DTX at 22,000 ×g for 30 min. Supernatant recovered from unloaded NPs (CH + CMD) was used as a blank. All measurements were done in triplicate. Finally, the loading efficiency percentage was calculated using the following formula:


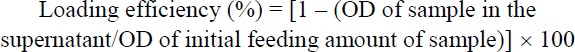


### Evaluation of *in vitro* drug and siRNA release

The release of siRNA and drug from loaded NPs (IGF-1R siRNA + CMD + CH and DTX + CMD + CH) was examined by incubating the NPs in phosphate buffer solutions (PBS, pH 7.4 and pH 5.5) at 37 °C. Briefly, the NPs were dispersed in 5 ml of PBS in a membrane dialysis bag (12 kDa cut-off, Merck^®^, USA). Then the dialysis bag was immersed in the beaker containing 50 ml of PBS (pH 7.4 and pH 5.5) and placed in a shaker incubator (37 °C, 94 rpm) for 120 h. Thereafter, at various time intervals, 2 ml of solutions were withdrawn and replaced with the same volume of fresh PBS under the same condition. Finally, siRNA and drug released contents were measured by UV-Vis spectrophotometer (Nanodrop ® 2000, Thermo Scientific, Worcester, MA, USA) at 260 and 230 nm, respectively. In addition, the release medium collected from unloaded NPs (CH + CMD) was used as a blank. *In vitro* drug and siRNA release (%) were calculated using the following formula:


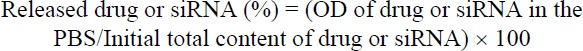


### Stability of siRNA-loaded NPs in serum and heparin

A volume of 400 µl of the siRNA-loaded NPs (containing 57 µg of siRNA) were incubated with a 200 µl of 10% FBS at 37 °C. At each time interval (2, 8, 12, 24, and 48 h), 40 µl of the mixture was withdrawn and stored at -20 °C until gel electrophoresis was performed. The naked siRNA was used as the control. For the evaluation of the stability of siRNA-loaded NPs in heparin, 60 µl of siRNA-loaded NPs were incubated with 2 µg/ml of heparin in different volumes (0, 0.6, 1.5, and 3 µl) at 37 °C for 1 h. Finally, the stability was analyzed by gel electrophoresis.

### Synthesis of Apt-conjugated NPs

#### EDC/NHS crosslinking method

The conjugation of Apt to NPs was performed using the previously described method[16]. Briefly, NPs were suspended in 200 µl of DNase/RNase-free water and mixed with EDC (10 mg) and NHS (8 mg) at room temperature for 2 h. For removal of the unreacted EDC and NHS, membrane dialysis bag (12 kDa cut-off, Merck^®^) was used. Thereafter, 5’-NH2-modified MUC1 Apt (1% w/w) was reacted with activated NPs at room temperature for 8 h. Finally, MUC1 Apt-conjugated NPs were purified by two successive centrifugation steps (16,000 ×g, 5 °C, 10 min).

#### Confirmation of Apt-NPs conjugation

Agarose gel electrophoresis (2% w/v) in 1 M Tris-acetate-EDTA (TEA buffer) solution was performed to confirm the conjugation of MUC1 Apt to NPs in different groups (free Apt, unpurified Apt-conjugated NPs, and purified Apt-conjugated NPs).

### Evaluation of cellular uptake

The cellular uptake of Apt-conjugated NPs by SKBR3 and CHO cells was studied using Cell Imaging Multi-Mode Reader (Cytation™ 5, BioTek, USA). Apt-unconjugated NPs were used as non-targeted NPs. Cells were seeded in six-well plates under standard culture conditions (RPMI-1640 culture medium supplemented with 10% FBS, 100 IU of penicillin, and 100 µg/ml of streptomycin) with 5% CO_2_ and 95% relative humidity at 37°C for 24 h. The medium was removed and cells were treated with Apt-conjugated NPs and Apt-unconjugated NPs containing 2 µg/µl of GFP plasmid for 24 h. GFP plasmid NPs were prepared based on siRNA-loaded NPs as described above. Thereafter, the cells were washed with PBS (pH 7.4) three times and fixed with 4% formaldehyde at room temperature for 30 min. The fixed cells were incubated with DAPI (nucleic acid stain) for 5 min. Finally, the transfected cells were evaluated using Cytation™ 5 for their GFP expression and were quantified by ImageJ software (http://rsbweb.nih.gov/ij/).

### MTT assay

The cytotoxicity and IC50 values of free DTX and NPs-loaded DTX were previously assessed[11,17]. Therefore, the impact of treatment groups on cell viability of SKBR3 cells was evaluated using MTT assay. The cells (1 × 10^4^/well) were seeded on 96-well plates under standard culture conditions as mentioned before. After complete cellular attachment, the culture medium was first removed and substituted with a fresh medium. The cells were treated with different pharmaceutical groups for 24 and 48 h in the separated plates. Afterwards, 100 µl of MTT solution (5 mg/ml) and an equal volume of culture medium were added to each well. The aforementioned solution was removed after 3-4 h, and formazan crystals were dissolved in 200 µl of DMSO and 25 µl of Sorenson’s buffer at 25 °C for 20 min. OD was read at 492 nm versus 690 nm reference wavelength using an ELISA reader (Stat Fax 2100, USA). All experiments were performed in triplicate. Finally, cell viability (%) was calculated using the following equation:


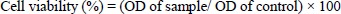


### RNA extraction and cDNA synthesis

The SKBR3 cells (5 × 10^5^ cells/well) were seeded on six-well plate under standard cell culture condition as mentioned before. The cells were treated with different pharmaceutical groups for 24 and 48 h in the separated plates. RNA extraction was performed using RNX-plus^®^ solution according to the manufacturer’s guideline. The purity of RNA was assayed by UV-vis spectrophotometer (Nanodrop^®^ 2000, USA), and samples were stored at -70 °C for further evaluations. cDNA was synthesized with 1 µl of total RNA (5 ng), 1 µl of oligodT, 1 µL of the random hexamer, 4 µl of reverse transcriptase buffer (5×), 0.5 µl of reverse transcriptase, and 2 µl of the deoxynucleotide triphosphates mix.

### Quantitative real-time PCR (qRT-PCR)

The qRT-PCR method was performed using the SYBR Green Real-time PCR master mix (Ampliqon^®^, Denmark) with a total volume of 10 μL on the Roche LightCycler^®^ 96 System. Primers, as shown in Table 1, were designed with OLIGO 7 analysis software. The thermal cycles were: denaturation at 94 °C for 10 min, amplification for 45 cycles of 10 s at 94 °C, annealing at 60 °C for 30 s and a final extension of 10 s at 72°C. The relative mRNA levels were calculated by 2^−ΔΔCT^ method, using 18S rRNA as a housekeeping gene.

**Table 1:** Sequences of primers used in this study

Genes	Sequences
*18s rRNA*	F:5´-GCATAGATAGCCGTCGTAGTTCC-3´
	R: 5´-CTGTCAATCCTTCCGTGTC-3´
*STAT3*	F: 5´-AGTTTCTGGCCCCTTGGATTG-3´
	R: 5´-CAGGAAGCGGCTATACTGCTG-3´
*MMP-9*	F: 5´-ATTCATCTTCCAAGGCCAATCC-3´
	R: 5´-CTTGTCGCTGTCAAAGTTCG-3´
*VEGF*	F: 5´-TCACCAAGGCCAGCACATAG-3´
	R: 5´-GACAGCAGCGGGCACCAAC-3´
*IGF-1R*	F: 5′ -TCTGGCTTGATTGGTCTGGC-3′
	R: 5′-AACCATTGGCTGTGCAGTCA-3′

### Statistical analysis

Statistical analysis was performed via GraphPad Prism 6.0 (GraphPad Software, La Jolla, CA, USA). The results were evaluated using one-way ANOVA and Mann-Whitney U test as appropriate. Probability values of less than 0.05 were considered to be statistically significant. The results presented in the text and tables represent mean ± standard deviation (SD).

## RESULTS

### Characterization of NPs

**Fig. 1:**
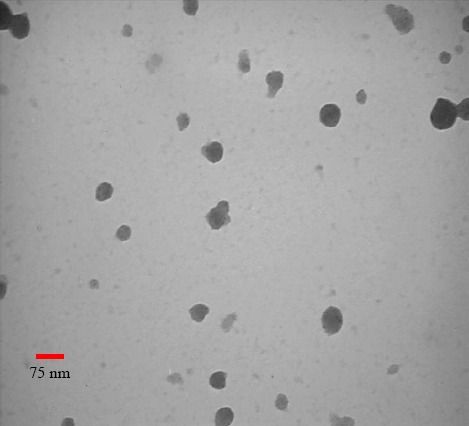
The morphology of nanodrugs.

**Fig. 2:**
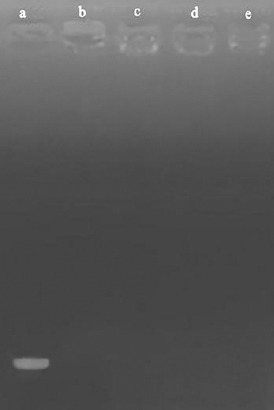
siRNA loading test. Lane a, naked siRNA; lane b, CH + CMD + siRNA (28.5 µg); lane c, CH + CMD + siRNA (57 µg); lane d, CH + CMD + siRNA (85.5 µg); lane e, CH.

The particle size, PDI, and zeta potential of nano-drugs are shown in Table 2. The smooth surface and spherical morphology of NPs were observed by TEM (Fig. 1). The complete entrapment of siRNA into 50-kDa CH-NPs is shown in Figure 2. Finally, the loading efficiency of siRNA and DTX were determined as 91.2 and 87.6%, respectively.

**Table 2:** The particle size, PDI, and zeta potential of nanodrugs

Characteristics\Nanodrug	Size (nm)	PDI	Zeta potential (mV)
DTX-siRNA-CH	110.2 ± 3.12	0.227 ± 0.02	14.3 ± 0.2
Apt (DTX-siRNA-CH)	118.4 ± 4.23	0.296 ± 0.03	12.2 ± 0.3

### Evaluation of *in vitro* drug and siRNA release

**Fig. 3:**
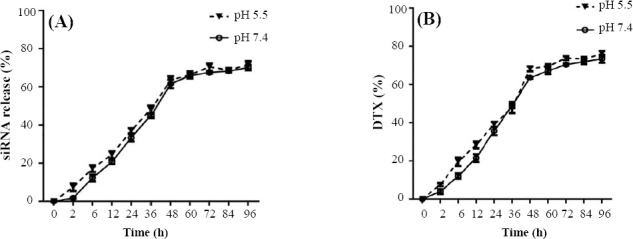
Drug release behavior. (A) siRNA release (%) from loaded NPs (IGF-R siRNA + CMD + CH) and (B) DTX release (%) from loaded NPs (DTX + CMD + CH) in PBS at different pHs (7.4 and 5.5).

The release pattern of DTX and IGF-1R siRNA at 37 °C at different pHs (7.4 and 5.5) is shown in Figure 3.

The slow release of IGF-1R siRNA was observed until 48 h in both pHs and reached the steady-state phase after 60 h. On the other hand, the slow release of DTX was observed until 60 h in both pHs and reached the steady-state phase after 72 h. Furthermore, the release pattern of IGF-1R siRNA and DTX in both pHs was not different. It was observed that CH-NPs released 50% of their contents (siRNA or DTX) in the first 36 h.

### Stability of siRNA-loaded NPs in serum and heparin

**Fig. 4:**
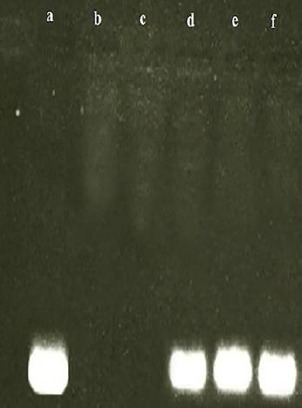
Stability test of NPs + siRNA at serum environment. Lane a, naked siRNA; lanes b, c, d, e, and f NPs + siRNA, 2, 8, 12, 24, and 48 h after exposure to FBS, respectively.

**Fig. 5:**
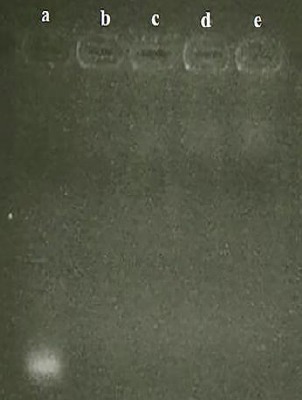
Stability test of NPs + siRNA at heparin environment. Lane a, naked siRNA (57 µg); lanes b, NPs + siRNA; lane c, NPs + siRNA + 0.6 µl heparin (2 µg/ml); lane d, NPs + siRNA + 1.5 µl heparin; lane e, NPs + siRNA + 3 µl heparin.

The incubation of IGF-1R siRNA-loaded NPs in serum and subsequent analysis through gel electrophoresis showed that the release of IGF-1R siRNA was at 12 h and continued until 48 h (Fig. 4). On the other hand, the IGF-1R siRNA-loaded NPs were stable in the presence of different concentrations of heparin (Fig. 5).

### Synthesis of Apt-conjugated NPs

**Fig. 6:**
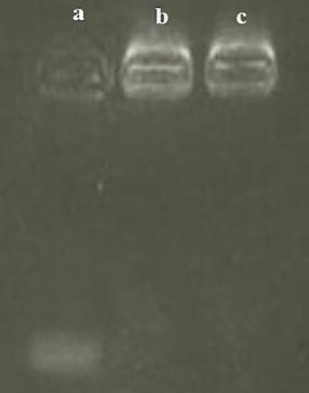
Confirmation of Apt conjugation. Lane a, unconjugated-MUC1 Apt; lane b, unpurified (NPs + Apt); lane c, purified NP + Apt.

Based on the results of agarose gel electrophoresis (Fig. 6), free MUC1 Apt showed a distinct band in the gel. However, the MUC1 Apt-conjugated NPs (before and after purification) showed no distinct bands. It seems that the whole of MUC1 Apt reacted with the activated NPs, and no distinct bands were observed in the gel of unpurified Apt-conjugated NPs (similar to the purified Apt-conjugated NPs).

### Evaluation of cellular uptake

**Fig. 7:**
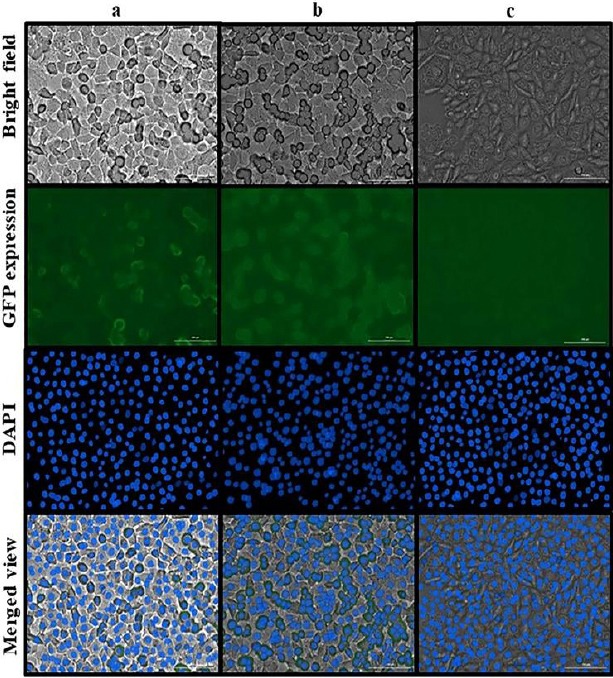
Evaluation of cellular uptake. (a) SKBR3 cells treated by NPs + GFP/plasmid; (b) SKBR3 cells treated by NPs + GFP/plasmid + Apt; (c) CHO cells treated by NPs + GFP/plasmid + Apt

The intensity of the GFP expression in the SKBR3 (MUC1 overexpressed cells) and CHO (MUC1 negative cells) was used as an indicator for the cellular uptake of Apt-unconjugated NPs and Apt-conjugated NPs. As it is apparent from Figure 7, the highest cellular uptake of NPs was observed in MUC1 Apt-conjugated NPs (GFP expression: 68%) in SKBR3 cells, but the lowest cellular uptake of MUC1 Apt-conjugated NPs was observed in CHO cells (GFP expression: 23%). The intensity of GFP expression in the SKBR3 cells, treated by Apt-unconjugated NPs, was 45%.

### Evaluation of cell viability

**Fig. 8:**
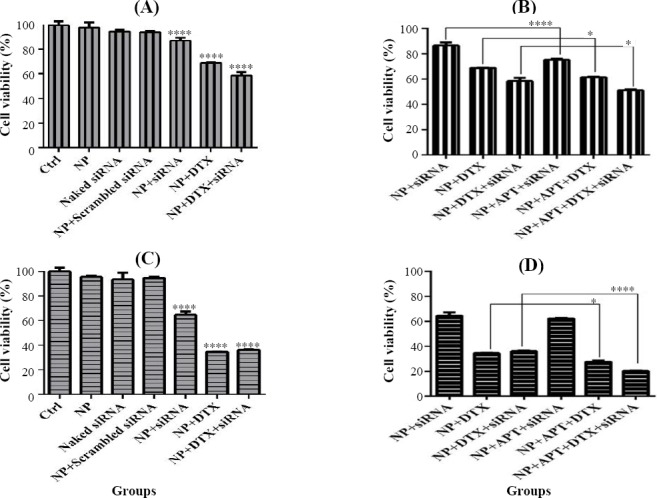
MTT assay. (A) MTT-24 h and (C) MTT-48 h, comparison of control (Ctrl) groups vs. treatment groups lacking MUC1 Apt; (B) MTT-24 h and (D) MTT-48 h, comparison of treatment groups lacking mucin1 aptamer vs. treatment groups containing MUC1 Apt (^*^p < 0.05 and ^****^p < 0.0001).

The MTT bioassay results indicated that NP-siRNA, NP-DTX, and NP-DTX-siRNA significantly (*p* < 0.0001) decreased the viability of SKBR3 cells after 24 and 48 h as compared with the control group. Furthermore, a significant decrease in the viability of SKBR3 cells was observed in all Apt-conjugated treatment groups, including NP-Apt-siRNA (*p* < 0.0001), NP-Apt-DTX (*p* < 0.05), and NP-Apt-DTX- siRNA (*p* < 0.05) after 24 h, as compared with the corresponding Apt-unconjugated treatment groups. In addition, NP-Apt-DTX (*p* < 0.05) and NP-Apt-DTX- siRNA (*p* < 0.0001) significantly reduced the viability of SKBR3 cells after 48 h when compared with the corresponding Apt-unconjugated treatment groups (Fig. 8).

### Gene expression studies

The qRT-PCR was performed to evaluate the effect of different pharmaceutical groups on the expression of the following genes: *IGF-1R*, *STAT3*, *MMP9*, and *VEGF*. All results were represented as mean expression fold ± SD.

### *IGF-1R* gene silencing

**Fig. 9:**
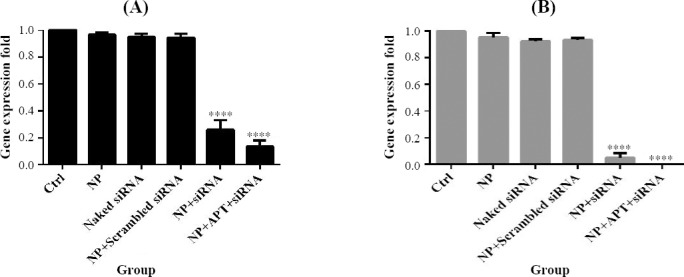
*IGF-1R* gene silencing. Gene expression pattern under 24 h (A) and 48 h (B) after treatment with different pharmaceutical groups (^****^p < 0.0001).

NP + siRNA (0.26 ± 0.071) and NP + APT + siRNA (0.134 ± 0.047) significantly (*p* < 0.0001) decreased the gene expression of *IGF-1R* in SKBR3 after 24 h in comparison to the control group. In addition, NP + siRNA (0.049 ± 0.033) and NP + APT + siRNA (0.001) significantly (*p* < 0.0001) decreased the gene expression of *IGF-1R* in SKBR3 after 48 h, as compared with the control group (Fig. 9).

### *STAT3* gene expression

**Fig. 10:**
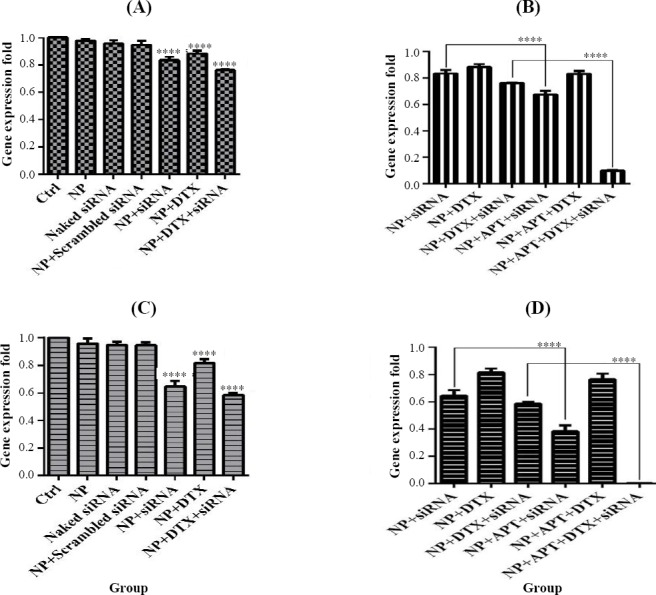
STAT3 gene expression assay. Gene expression pattern under 24 h and 48 h after treatment with MUC1 Apt lacking groups vs. control groups (A and C) and with MUC1 Apt containing groups vs. MUC1 Apt lacking groups (B and D); ^****^p < 0.0001.

NP + siRNA (0.833 ± 0.028; *p* < 0.0001), NP + DTX (0.883 ± 0.021; *p* < 0.001), and NP + DTX + siRNA (0.761 ± 0.003; *p* < 0.0001) significantly decreased the gene expression of *STAT3* in SKBR3 cells after 24 and after 48 h (0.646 ± 0.041, 0.816 ± 0.027, and 0.587±0.014, respectively; *p* < 0.0001), when compared with the control group. Also, NP + APT + siRNA (0.674 ± 0.029; *p* < 0.0001) and NP + APT + DTX+ siRNA (0.098 ± 0.005; *p* < 0.0001) significantly reduced the gene expression of *STAT3* after 24 and 48 h (0.383 ± 0.045 and 0.003 ± 0.001, respectively; *p* < 0.0001), in comparison with the corresponding Apt-unconjugated treatment groups. (Fig. 10).

### *MMP9* gene expression

**Fig. 11:**
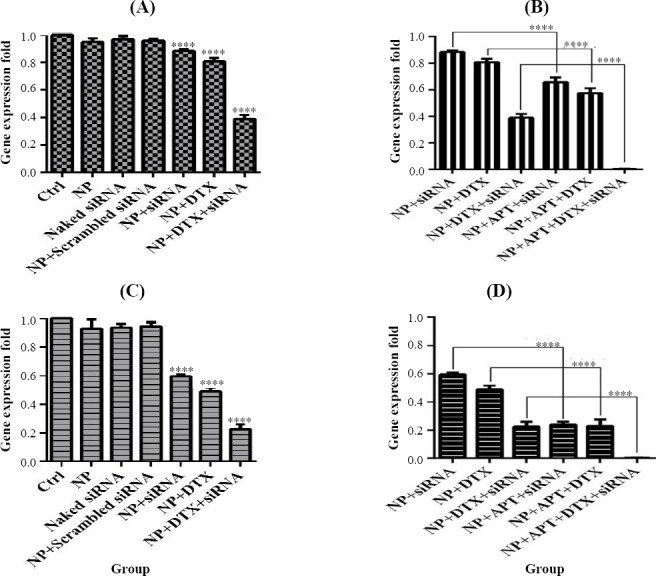
*MMP9* gene expression assay. Gene expression pattern under 24 h and 48 h after treatment with MUC1 Apt lacking groups vs. control groups (A and C) and MUC1 Apt containing groups vs. MUC1 Apt lacking groups (B and D); ****p < 0.0001.

NP + siRNA (0.885 ± 0.009; *p* < 0.001), NP + DTX (0.809 ± 0.026; *p* < 0.0001), and NP + DTX + siRNA (0.389 ± 0.02; *p* < 0.0001) significantly reduced the gene expression of MMP9 in SKBR3 cells after 24 h, as compared with the control group. In addition, NP + APT + siRNA (0.657 ± 0.036), NP + APT + DTX (0.574 ± 0.038), and NP + APT + DTX + siRNA (0.003 ± 0.0006) significantly decreased the gene expression of *MMP9* after 24 h (*p* < 0.0001), in comparison with the corresponding Apt-unconjugated treatment groups. Furthermore, as compared with the control group, NP + siRNA (0.595 ± 0.012), NP + DTX (0.488 ± 0.029), and NP + DTX + siRNA (0.224 ± 0.037) significantly decreased the gene expression of MMP9 after 48 h (*p* < 0.0001). However, NP + APT + siRNA (0.242 ± 0.017), NP + APT + DTX (0.229 ± 0.047), and NP + APT + DTX + siRNA (0.002 ± 0.0013) significantly decreased the gene expression of *MMP9* after 48 h (*p* < 0.0001), as compared with the corresponding Apt-unconjugated treatment groups (Fig. 11).

**Fig. 12:**
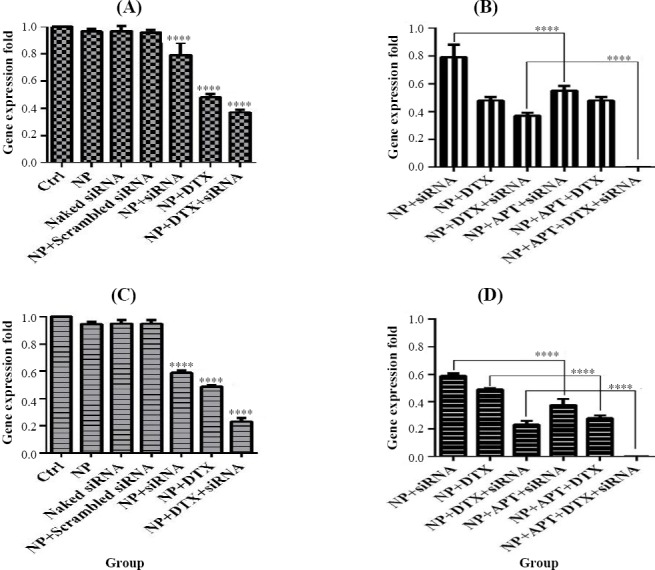
*VEGF* gene expression assay. Gene expression patterns after 24 h and 48 h of treatment with MUC1 Apt lacking groups vs. control groups (A and C) and MUC1 Apt with containing groups vs. MUC1 Apt lacking groups (B and D); ^****^p < 0.0001.

### *VEGF* gene expression

NP + siRNA (0.792 ± 0.09), NP + DTX (0.481 ± 0.024), and NP + DTX + siRNA (0.368 ± 0.023) significantly decreased the gene expression of *VEGF* in SKBR3 cells after 24 and 48 h (0.589 ± 0.017, 0.489 ± 0.009, and 0.232 ± 0.028, respectively)as compared with the control group (*p* < 0.0001). In addition, a significant decrease was observed in the gene expression of *VEGF* using NP + APT + siRNA (0.55 ± 0.035) and NP + APT + DTX + siRNA (0.002 ± 0.0004) after 24 and 48 h (0.375 ± 0.045, 0.279 ± 0.021, and 0.002 ± 0.0009), as compared with the corresponding Apt-unconjugated treatment groups (*p* < 0.0001).

## DISCUSSION

Treatment of breast cancer has become a complicated dilemma. One of the protocols to achieve the novel combination therapies is the utilization of drug delivery systems for the purpose of simultaneous delivery of chemotherapeutic agents and gene silencing molecules such as siRNA. The effectiveness of this treatment would be increased if it becomes more targeted through the addition of smart ligands, known as “aptamers”. In the present study, the MUC1 Apt-conjugated NPs were used as the suitable carriers of DTX and IGF-1R siRNA to evaluate the cell viability and genes expression on SKBR3 cells. Characterization of nanodrugs indicated the formation of smooth spherical NPs with positive zeta potential and a size of less than 150 nm. The advantage of spherical nanodrugs over rod-shaped particles is in the controlled release of the contents of NPs into the tumor cells. The positive zeta potential of nanodrugs is important for transferring siRNA with the least exposure to plasma endonucleases[15]. Also, the positive charge of NPs increases the loading efficiency of siRNA[18]. In other words, the addition of negative-charged polymer of CMD is useful for the prevention of excessive increase in nanodrugs’ positive zeta potential of more than +15 mV[19]. Also, CMD covers the NPs and protects them from degradation by phagocytic cells[15]. The results of our study confirmed the findings of other studies reporting that the conjugation of Apt to NPs would not considerably affect the size and zeta potential of nanodrugs[16,20]. Generally, the advantages of the designed NPs, including the appropriate size, shape, and zeta potential were in line with the above mentioned studies.

The molecular weight of CH affects the NPs size and loading efficiency of siRNA[3]. According to Jadidi-Niaragh *et al*.[21], the molecular weight of 50 kDa for CH is the most efficient for the treatment of tumor cells with the highest loading of siRNA into NPs. As for stability tests of siRNA-loaded NPs in exposure to heparin and serum, these NPs were stable in heparin solution, and the stability time in serum solution was 12 h. Research study conducted by this researcher has confirmed the results of our study regarding the stability of siRNA-loaded NPs in heparin solution, and the stability time in serum solution was reported to be up to 9 h[21]. However, the results of another study found a stability of 48 h in serum solution[22].

We used EDC and NHS to activate the conjugation sites of NPs to interact with the secondary amine groups of MUC1 Apt. The MUC1 Apt-conjugated NPs showed no clear band on the gel neither before nor after purification, suggesting that the conjugation of the whole molecules of Apt to NPS with no detectable remained free molecules of Apt. In the present study, the cellular uptake assay of MUC1 Apt-conjugated NPS, which was done on MUC1^-^ (CHO) and MUC1^+^ (SKBR3) cells, confirmed the role of Apt in targeted delivery. This finding is in agreement with other the reports of other studies[16,23].

In line with other studies[24,25], formulating DTX through CH NPS was safe because the naked NPS did not represent any considerable toxic effect on cell viability. This is one of the advantages of the CH-NPs due to the toxicity of docetaxel. A combination of DTX and siRNA in comparison with monotherapy using DTX showed better results on cell viability. On the other hand, making this combination treatment targeted via Apts has improved the results.

STAT3 is a crucial factor in tumor progression and metastasis via the up-regulation of downstream genes such as *MMPs* and *VEGF*[26,27]. Although the activation of IGF/IGF-1R signaling axis was not the only pathway that activated STAT3, as shown in the present study, the complete nano-drug (NP + APT + siRNA + DTX) dramatically reduced the expression of *STAT3* gene. This result may be attributed to the synergistic effects of DTX and IGF-1R siRNA and also Apt dependent targeted co-delivery. In a study by Subramani *et al*.[7], it has been demonstrated that IGF-1R silencing significantly decreased the activated form of STAT3 in pancreatic cancer cells. In addition, the down-regulation of *STAT3* gene may be effective in the prevention of DTX resistance in cancer therapy[28]. MMPs, such as MMP9, are important in destroying the extracellular matrix barrier via the degradation of type IV collagens and assisting the tumor cell invasion and metastasis. The overexpression of MMP9 has previously been demonstrated in breast cancer. Moreover, the low expression of MMP9 is a good indicator of a patients’ prognosis[29]. As shown in the present study, nano-formulation of IGF-1R siRNA or DTX and the combined form of nanodrug (NP + siRNA + DTX) were able to significantly decrease the gene expression of *MMP9*. Furthermore, the significant decrease in the gene expression of *MMP9* was obtained when the Apt was conjugated to nanodrug as compared with Apt-unconjugated complete group. Qian *et al*.[30] have also revealed that IGF-1R knock-down reduced the gene expression of MMP9 in lung cancer cells and invasion of tumor cells in an animal model. In another study, the inhibitory effect of non-cytotoxic concentrations of free DTX on down-regulation of MMP9 was observed in colorectal cancer cells[31].

The pivotal role of VEGF in tumor angiogenesis has been extensively studied, and high expression level of VEGF has been shown to be associated with poor prognosis of breast cancer[32]. According to the results of the present study, the significant decrease in *VEGF* gene expression, similar to the *MMP9* gene expression, was observed when Apt was conjugated to the NP + siRNA, NP + DTX, and NP + DTX + siRNA. Yeo *et al*.[33] have demonstrated that IGF-1R tyrosine kinase inhibitor, linsitinib, inhibited VEGF in non-small cell lung cancer cells. Moreover, the non-cytotoxic concentration of DTX could decrease the expression of VEGF in colorectal cancer cells[31]. In addition, there was a correlation between IGF-1R signaling pathway and MUC1 expression in breast cancer cells. IGF-1R up-regulates MUC1 expression in breast cancer cells in a PI3K/Akt signaling pathway-dependent manner and subsequently promotes epithelial-mesenchymal transition that is important in metastasis. Therefore, IGF-1R knock-down can prevent the up-regulation of MUC1 and epithelial-mesenchymal transition in cancer cells[34]. Consequently, targeted co-delivery of IGF-1R siRNA and DTX may enhance the therapeutic properties of nanodrugs.

In this study, the MUC1 Apt-conjugated CH NPs for the co-delivery of DTX and IGF-1R siRNA to SKBR3 cells were designed. We demonstrated that this novel targeted co-delivery system could enhance the cellular uptake of NPs and profoundly decreased the cell viability and genes expression involved in the progression of tumors and metastasis. Considering the appropriate pharmaceutical and *in vitro* pharmacological properties of the designed nanodrug, animal model studies need to be conducted to confirm the efficacy and safety of this nanodrug.
